# Supplementary material for: A model-based approach for detecting and identifying faults on the D.C. side of a P.V. system using electrical signatures from I-V characteristics
Source: PLoS One. 2022 Mar 29;17(3):e0260771. doi: 10.1371/journal.pone.0260771 (PMC8963553; doi:10.1371/journal.pone.0260771)
Supplement: S1 File — (DOCX) [file pone.0260771.s002.docx]

**Comments on Data set**

Dear Dr. Khan,

We've checked your submission and before we can proceed, we need you to address the following issues:

1. Thank you for providing a supporting information data file. At this time, please confirm that your submission contains your "minimal data set", which PLOS defines as consisting of the data set used to reach the conclusions drawn in the manuscript with related metadata and methods, and any additional data required to replicate the reported study findings in their entirety. This includes:

1. The values behind the means, standard deviations and other measures reported.
2. The values used to build graphs.
3. The points extracted from images for analysis.

**Response To Above Comments**

I would like to respond to queries about the data file I have shared with the journal's paper review team upon request.

1. First, Team has asked to confirm whether the submission contains a minimal data set, which PLOS defines as consisting of the data set used to reach the conclusions drawn in the manuscript with related metadata and methods.

Author would like to respond "Yes" with a little elaboration on data submitted, as below.

i) The submission contains the source code used for finding three points on the I-V curve i.e., short circuit point,
ii) open circuit point and
iii) maximum power point.

Each time a condition was simulated on the Simulink model, the corresponding code was run afterwards in MATLAB which used to calculate values of those three points and return them to Simulink for further calculation of derived parameters from those three points. This is the first step towards finding I-V characteristics and calculating from them the parameters required for creating electrical signatures.

ii) The parameters were calculated from those three points in Simulink by their corresponding models and that's why no code for their calculations needed. Values of those parameters were noted down from their Simulink models. Then, a code was written for the sake of obtaining graphs for the parameters' data, as graphical representation best represents a set a data for comparison purposes. That plotting parameters' data code contains crucial data regarding results and that's why code along with data for only two parameters was provided in the submission.

2. Secondly, it was asked if it contains additional data such as the values behind the means, standard deviations and other measures reported, values used to build graphs and the points extracted from images for analysis.

The answer to this question is that the data file contains the MATLAB/Simulink generated simulation workspace data for each condition applied on the system, in form arrays. Uploading that .mat file to MATLAB workspace brings all I-V and P-V data obtained for various conditions. Then, a code was written in the script for the purpose of obtaining I-V and P-V graphs for each applied condition. That code is also provided in the submission.

Furthermore, there was written any code for obtaining parameters such as standard deviations and mean. Rather, an extensive analysis was carried out and proper limits for deviations from normal value were set such that they help in better creation of diagnostic algorithm. Diagnostic algorithm flow chart is already provided in the manuscript and no such code was written for it as no real test was conducted to check its application. The algorithm was obtained based on analysis of parameters response towards different faults.
