# Supplementary material for: A model-based approach for detecting and identifying faults on the D.C. side of a P.V. system using electrical signatures from I-V characteristics
Source: PLoS One. 2022 Mar 29;17(3):e0260771. doi: 10.1371/journal.pone.0260771 (PMC8963553; doi:10.1371/journal.pone.0260771)
Supplement: Attachment — Submitted filename: Response to Reviewers.docx [file pone.0260771.s003.docx]

**Original Manuscript ID**: PONE-D-21-20995R1

**Original Article Title: “A model-based approach for detecting and identifying faults on the D.C. side of a P.V. system using electrical signatures from I-V characteristics**”

**To:** The Editor PLOS ONE

**Re:** Response to reviewers

Dear Editor,

Thank you for allowing a resubmission of our manuscript, with an opportunity to address the reviewers’ comments.

We are uploading (a) A rebuttal letter that responds to each point raised by the academic editor and reviewer(s) send as file labelled 'Response to Reviewers'. (b) A marked-up copy of the manuscript that highlights changes made to the original version, labelled 'Revised Manuscript with Track Changes (Yellow Highlighted)'. (c) An unmarked version of the revised paper without tracked changes, labelled 'Manuscript'.

The authors received no specific funding for this work.

Best regards,

**Khalid Khan et al.**

**Editor’s comments,**

**Comments to the Author**

Thank you for submitting your manuscript to PLOS ONE. After careful consideration, we feel that it has merit but does not fully meet PLOS ONE’s publication criteria as it currently stands. Therefore, we invite you to submit a revised version of the manuscript that addresses the points raised during the review process. We look forward to receiving your revised manuscript.

1. Please ensure that your manuscript meets PLOS ONE's style requirements, including those for file naming. The PLOS ONE style templates can be found at

<https://journals.plos.org/plosone/s/file?id=wjVg/PLOSOne_formatting_sample_main_body.pdf> and <https://journals.plos.org/plosone/s/file?id=ba62/PLOSOne_formatting_sample_title_authors_affiliations.pdf>

**Author’s response:** The corresponding author, on behalf of all the co-authors, would like to thank the editor for the positive feedback and encouragement. The revised version of the manuscript has been updated according to the guidelines provided by the PLOS ONE style requirements.

2. Thank you for stating the following financial disclosure:

**Author’s response:** The authors received no specific funding for this work.

a) Please clarify the sources of funding (financial or material support) for your study. List the grants or organizations that supported your study, including funding received from your institution.

**Author’s response:** No funding or any financial grant has been received for conducting this study.

b) State what role the funders took in the study. If the funders had no role in your study, please state:

**Author’s response:** The authors received no specific funding for this work.

c) If any authors received a salary from any of your funders, please state which authors and which funders.

**Author’s response:** The authors received no specific funding for this work.

3. We note that you have indicated that data from this study are available upon request. PLOS only allows data to be available upon request if there are legal or ethical restrictions on sharing data publicly. For more information on unacceptable data access restrictions, please see <http://journals.plos.org/plosone/s/data-availability#loc-unacceptable-data-access-restrictions>.

**Author’s response:** A source file of the code is shared as a zip file in the review submitted.

**In your revised cover letter, please address the following prompts:**

a) If there are ethical or legal restrictions on sharing a de-identified data set, please explain them in detail (e.g., data contain potentially sensitive information, data are owned by a third-party organization, etc.) and who has imposed them (e.g., an ethics committee). Please also provide contact information for a data access committee, ethics committee, or other institutional body to which data requests may be sent.

**Author’s response:** There are no ethical restrictions on sharing a de-identified data set.

b) If there are no restrictions, please upload the minimal anonymized data set necessary to replicate your study findings as either Supporting Information files or to a stable, public repository and provide us with the relevant URLs, DOIs, or accession numbers. For a list of acceptable repositories, please see <http://journals.plos.org/plosone/s/data-availability#loc-recommended-repositories>.

**Author’s response:** The data set is uploaded in the review as a Supporting Information file.

4. Please amend your list of authors on the manuscript to ensure that each author is linked to an affiliation.

**Author’s response:** The list of authors is provided with affiliation in the given order, which is requested to be considered in the revised version of the manuscript.

i. **Muhammad Adnan Khan^1^,**

Affiliation: Research Associate, Center for Advanced Studies in Energy University of Engineering and Technology, Phase 5, opposite to Sui Northern Gas Pipeline office, Postal address 25000, Peshawar.

ii. **Khalid Khan^2*^^[1]^**

Affiliation: Research Associate, Center for Advanced Studies in Energy University of Engineering and Technology, Phase 5, opposite to Sui Northern Gas Pipeline office, Postal address 25000, Peshawar.

iii. **Adnan Daud Khan^3*^**

Affiliation: Dean Faculty Renewable Energy, Center for Advanced Studies in Energy University of Engineering and Technology, Phase 5, opposite to Sui Northern Gas Pipeline office, Postal address 25000, Peshawar.

iv. **Zubair Ahmad Khan^4^**

Affiliation: Professor at Department of Mechatronics, University of Engineering and Technology, Phase 5, opposite to Sui Northern Gas Pipeline office, Postal address 25000, Peshawar.

v. **Shahbaz Khan^5^**

Affiliation: Lab Engineer at Department of Mechatronics, University of Engineering and Technology, Phase 5, opposite to Sui Northern Gas Pipeline office, Postal address 25000, Peshawar.

vi. **Muhammad Rizwan Siddiqui^6^**

Lecturer at Capital University of Science and technology (CUST), Islamabad Expressway, Kahuta، Road Zone-V Sihala, Islamabad, Islamabad Capital Territory.

5. PLOS authors have the option to publish the peer review history of their article. If published, this will include your full peer review and any attached files.

**Author’s response:** The authors agree to publish the review history of the article.

**Please carefully address the comments of two reviewers to improve your paper.**

**Editor’s comments,**

**Concern #1:**

Is the manuscript technically sound, and do the data support the conclusions?
The manuscript must describe a technically sound piece of scientific research with data that supports the conclusions. Experiments must have been conducted rigorously, with appropriate controls, replication, and sample sizes. The conclusions must be drawn appropriately based on the data presented.

**Reviewer #1**: Partly

**Author response:** The authors appreciate the positive remarks of the reviewer #1 and would like to reflect on any particular section of the manuscript to improve its quality, if highlighted by the reviewer.

**Reviewer #2:** Yes

**Author response:** The authors appreciate the positive response of the reviewer #2.

**Concern #2:**

Has the statistical analysis been performed appropriately and rigorously?

**Reviewer #1:** I Don't Know

**Author response:** The authors would be happy to elaborate further on the techniques used in the article, provided specific questions are asked to be addressed.

**Reviewer #2:** Yes

**Author response:** The authors appreciate the feedback of the reviewer #2.

**Concern #3:**

Have the authors made all data underlying the findings in their manuscript fully available?

The [PLOS Data policy](http://www.plosone.org/static/policies.action#sharing) requires authors to make all data underlying the findings described in their manuscript fully available without restriction, with rare exception (please refer to the Data Availability Statement in the manuscript PDF file). The data should be provided as part of the manuscript or its supporting information or deposited to a public repository. For example, in addition to summary statistics, the data points behind means, medians and variance measures should be available. If there are restrictions on publicly sharing data—e.g., participant privacy or use of data from a third party—those must be specified.

**Reviewer #1:** Yes

**Author response:** The authors appreciate the positive feedback of the reviewer #1.

**Reviewer #2:** Yes

**Author response:** The authors appreciate the positive feedback of the reviewer #2.

**Concern #4:**

Is the manuscript presented in an intelligible fashion and written in standard English?

PLOS ONE does not copyedit accepted manuscripts, so the language in submitted articles must be clear, correct, and unambiguous. Any typographical or grammatical errors should be corrected at revision, so please note any specific errors here.

**Reviewer #1:** Yes

**Author response:** The authors appreciate the positive feedback of the reviewer #1.

**Reviewer #2:** Yes

**Author response:** The authors appreciate the positive response of the reviewer #2.

**5. Review Comments to the Author**

Please use the space provided to explain your answers to the questions above. You may also include additional comments for the author, including concerns about dual publication, research ethics, or publication ethics.

**Reviewer #1:**

1. There are places in the article that do not indicate which equation to cite, such as the paragraph above Equation 7.

**Author’s response:** The author appreciates reviewer #1 comment on this issue, as it was mistakenly left by author in the earlier draft. This mistake has been rectified and all equations are numbered properly as well as cited properly in the new draft. Equations after correction are highlighted yellow in the new draft.

2. What does P.S. (3.1) mean in the descriptive paragraph of Figure 6?

**Author’s response:** This question has been answered more properly and the issue has been addressed in the new draft in the last paragraph of methodology section, being highlighted in yellow there. Hope this will answer reviewer’s question.

3. In the analysis of Figure 6, it is mentioned that four different regions can be set for values beyond the predefined thresholds, but it is not clearly described what the four regions are and how to define the boundary between the four regions.

**Author’s response:** To answer this question, a brief description has been added to the new draft just below fig. (6), in highlighted text. This description has elaborated, in a brief way, on threshold levels and how they were selected. Furthermore, all the four regions regarding “Vte” are described and the boundaries between them are clearly defined in that. However, the focus of this paper is on checking the sensitivity of parameters that could serve in creating electrical signatures. Hence, it was found that Vte does the job by responding to various operating conditions in ways different enough from each other to differentiate between faults, which description tells clearly.

4. Appropriately add some relevant descriptions on how to determine the threshold range.

**Author’s response:** This question has already been answered in question 3. Apart from that, threshold levels are defined more clearly in first paragraph of Methodology section, text highlighted in yellow. Hope that will answer the question.

5. Add some comparisons with other scholars' work on electrical methods.

**Author’s response:** A comparison with other scholars’ work has been made at the end of results and discussions section. Highlighted in yellow.

6. Further elaborate on the specific advantages or innovations of this method.

**Author’s response:** It has been answered in the same description where comparison with other scholars’ work has been made, as stated above.

**Reviewer #2:**

1. The introduction of this submission should be improved further. The key differences between your work and previous studies should be clarified. A point-to-point way is recommended to state the main contributions.

**Author’s response:** The paper has been reviewed by the author after reviewers’ comments and the content has been improved. Hope it will satisfy the reviewer.

2. PV modelling section is very traditional, and this reviewer suggests this section can be shortened properly.

**Author’s response:** Author appreciate reviewer’s comment on PV modelling. In response, it is stated that PV modelling contains equations for modelling the related text to describe the terms used in model equations. No extra content has been added and author has aimed to contain modelling section in as small portion as possible.

3. The writing style of this work likes a technical report, but not a scientific paper.

**Author’s response:** Paper has been revised with minor changes as per reviewers’ comments. Hopefully the new draft resembles more like paper.

4. A comparison of your method and other work should be carried out.

**Author’s response:** Done at the end of Results and Discussion chapter.

5. Most of the figures have poor quality, such as, lacking unit.

**Author’s response:** This issue has been addressed in the new draft, where care has been taken while generating new figures for the paper.

**We are grateful to the editor and reviewers for the very constructive feedback. The manuscript has been thoroughly revised considering feedback provided by reviewers. We have tried to argue more effectively on the superiority of our proposed method. Further, the language of the manuscript has been thoroughly revised for an issue, typo, and other grammatical mistakes.**

1. Corresponding author: Engrkhaliduet@gmail.com
